# Plasticity of the MAPK Signaling Network in Response to Mechanical Stress

**DOI:** 10.1371/journal.pone.0101963

**Published:** 2014-07-15

**Authors:** Andrea M. Pereira, Cicerone Tudor, Philippe-Alexandre Pouille, Shashank Shekhar, Johannes S. Kanger, Vinod Subramaniam, Enrique Martín-Blanco

**Affiliations:** 1 Instituto de Biología Molecular de Barcelona (CSIC), Parc Cientific de Barcelona, Baldiri Reixac 10–12, Barcelona, Spain; 2 Nanobiophysics, MESA+ Institute for Nanotechnology & MIRA Institute for Biomedical Technology and Technical Medicine, University of Twente, Enschede, The Netherlands; Universitat Pompeu Fabra, Spain

## Abstract

Cells display versatile responses to mechanical inputs and recent studies have identified the mitogen-activated protein kinase (MAPK) cascades mediating the biological effects observed upon mechanical stimulation. Although, MAPK pathways can act insulated from each other, several mechanisms facilitate the crosstalk between the components of these cascades. Yet, the combinatorial complexity of potential molecular interactions between these elements have prevented the understanding of their concerted functions. To analyze the plasticity of the MAPK signaling network in response to mechanical stress we performed a non-saturating epistatic screen in resting and stretched conditions employing as readout a JNK responsive dJun-FRET biosensor. By knocking down MAPKs, and JNK pathway regulators, singly or in pairs in *Drosophila* S2R+ cells, we have uncovered unexpected regulatory links between JNK cascade kinases, Rho GTPases, MAPKs and the JNK phosphatase Puc. These relationships have been integrated in a system network model at equilibrium accounting for all experimentally validated interactions. This model allows predicting the global reaction of the network to its modulation in response to mechanical stress. It also highlights its context-dependent sensitivity.

## Introduction

Cells display versatile responses to mechanical inputs triggering signals leading to increased gene expression, protein synthesis, or mitogenesis [1], [2]. Altering the mechanical properties of the environment or subjecting cells to mechanical insults directs alternative differentiation pathways, promoting cytoskeleton rearrangements or altering the composition of the extracellular matrix [3]. For example, stem cells specification has been shown to be strongly influenced by the mechanical properties of the surrounding matrix [4].

Recent studies have identified some intracellular pathways mediating the biological effects observed upon mechanical stimulation. These include the mitogen-activated protein kinase (MAPK) cascades [5]. Three main MAPK pathways have been identified: the extracellular signal-regulated kinase (ERK), the c-Jun N-terminal kinase (JNK) and the p38 cascades. MAPK cascades are organized as modular pathways in which activation of upstream kinases leads to sequential activation of a MAPK module (MAPKKK – MAPKK - MAPK) [6]. Within these cascades, specificity is maintained primarily through structural mechanisms that limit protein interactions [7]. Further, all tiers of MAPK signaling can be regulated by protein phosphatases [8] emphasizing the importance of the balance between phosphorylation and dephosphorylation in regulating their functions. Finally, members of the Ras family of GTPases, including Ras itself and the Rho subfamily members, Rho, Rac1 and cdc42 trigger the activation of MAPK signaling. While Ras mainly targets the ERK pathway, Rho, Rac1 and cdc42 are mainly involved in the activation of JNK and p38 cascades [9], [10].

Conventionally, MAPK cascades have been depicted as linear signaling pathways insulated from each other. However, undefined mechanisms facilitating their crosstalk are also known to exist [11]. Indeed, recently published work indicates that ERK activity can be suppressed by JNK/p38 kinases through the activation of inhibitory phosphatases (PP2A, MKPs) [12], [13]. Yet, despite these extensive evidences, how different integrative cellular responses mediated by these cascades are regulated remains unknown. This is at least in part due to the combinatorial complexity of molecular interactions and a variety of feedback and feed-forward loops [14].

To analyze the plasticity of the MAPK network in response to mechanical stress we performed a non-saturating epistatic screen in resting and stretched conditions. To simplify the analysis we focused on the response of *Drosophila* S2R+ cells to stretch employing as readout the JNK responsive dJun-FRET biosensor previously shown to be a sensitive reporter of the activation of the pathway by mechanical stretch [15].

The genome of *Drosophila* possesses a single ERK encoded by the gene *rolled* (*rl*), a single JNK [*basket* (*bsk*)] and two p38 kinases [*mpk2* (*p38α*) and *p38β*] [16], [17]. It also contains a JNKK [*hemipterous* (*hep*)], a JN3K [*slipper* (*slpr*)], a JN4K [*misshapen* (*msn*)] and a JNK dual-specificity phosphatase [*puckered* (*puc*)] [18], [19]]. Several Rho family members have also been identified in *Drosophila* [20]. Further, several other MAPKK, MAP3K, MAP4K, MAPK phosphatases and three Ras homologues are also present in flies [21].

By knocking down MAPKs, and JNK pathway regulators, singly or in pairs by RNA interference (RNAi) in S2R+ cells, we have uncovered unexpected regulatory links between JNK cascade kinases, Rho GTPases, MAPKs and the JNK phosphatase Puc. These relationships are highlighted in a system network model at equilibrium accounting for the experimentally identified interactions. This model allows predicting the global reaction of the network to disparate inputs and, in particular, its modulation in response to mechanical stress. It also highlights its context-dependent sensitivity.

## Results

### Different roles for upstream dJun kinases in the mechanical stretch activation of dJun

We assessed in real-time the activity of the MAPK network in S2R+ cells by Fluorescence Lifetime Imaging Microscopy (FLIM) using an intramolecular phosphorylation-dependent dJun-FRET (Fluorescence Resonance Energy Transfer) biosensor. This biosensor has been shown in *Drosophila* BG2 cells to respond specifically to JNK activity [22]. Further, known chemical activators of the JNK pathway yield to a positive response of the dJun-FRET biosensor in *Drosophila* S2R+ cells that correlates with increased P-Jun levels [15]. These cells subjected to static mechanical stretch also show a significant increase in dJun-FRET biosensor response, reaching a stable steady state in approximately 20 minutes, which lasts for at least 3 hours [15].

To elucidate the contribution of individual JNK cascade elements in modulating the response of the dJun-FRET biosensor at rest or in the presence of mechanical stretch, we targeted *bsk*, *hep*, *slpr* and *msn* co-transfecting individual dsRNAs with the biosensor into S2R+ cells (see Methods).

**Figure 1:**
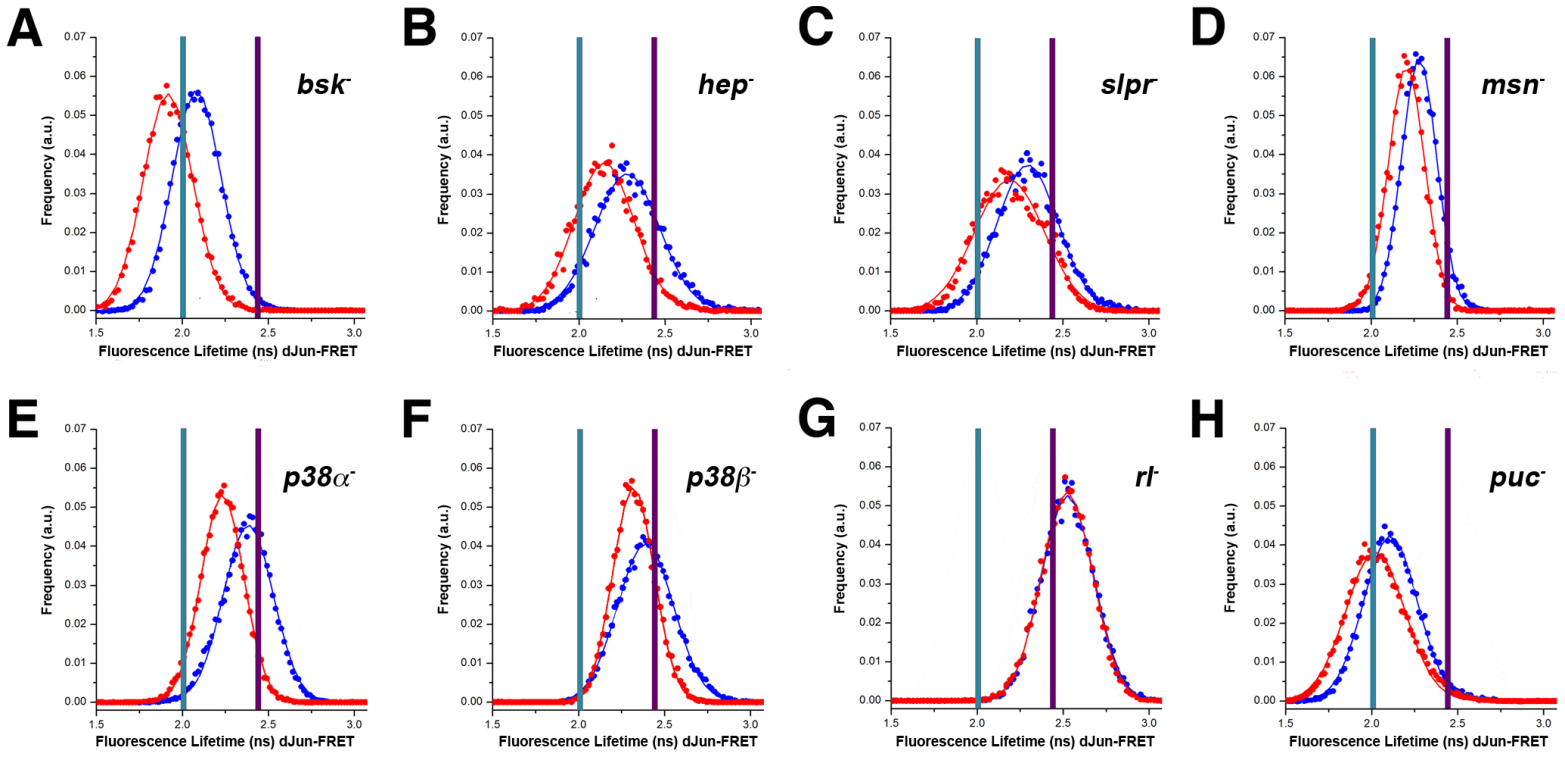
Distinct roles for kinases and phosphatases during mechanical stretch activation. *Drosophila* S2R+ cells were co-transfected with the pAct-dJun-FRET and different dsRNAs. **A**) *bsk^−^*. **B**) *hep^−^*. **C**) *slpr^−^*. **D**) *msn^−^*. **E**) *p38α^−^*. **F**) *p38β^−^*. **G**) *rl^−^*. **H**) *puc^−^*. Fluorescence lifetimes (FL) for the donor mCFP were collected and curves representing data recorded from ∼75 cells for cells at rest. Blue data points denote the measurements obtained at rest while red data points show the measurements obtained after 3 hours of continuous static stretch. In each panel, the purple bar represents the average FL determined for control wild type cells at rest, while the cyan bar represents the average FL of control wild type cells stretched for 3 hours.

At rest, the S2R+ cells treated with single dsRNAs against *bsk, hep, slpr* and *msn* show average FL values of 2.08±0.14 ns, 2.27±0.18 ns, 2.30±0.18 ns and 2.27±0.10 ns respectively (Figure 1A to D and Table S1). These lifetimes were significantly lower than the donor mCFP lifetime at rest (2.43±0.15 ns) for untreated cells (WT^R^) or cells treated with dsRNA against GFP. Summarizing, Bsk and its upstream kinases in S2R+ cells, unexpectedly and unlike in BG2 cells [22], behave as inhibitors of the biosensor in resting conditions, inhibition being the highest for Bsk.

As previously reported [15], static mechanical stretch of S2R+ cells for 2 hours resulted in a strong activation of the dJun-FRET biosensor, decreasing its FL to 2.00±0.15 ns (WT^S^), an average decrease of 0.43 ns versus WT^R^. Knocking down the JNK cascade kinases also altered the response of S2R+ cells to stretch. *bsk^−^* stretched cells showed average FL values of 1.91±0.14 ns, while *hep^−^, slpr^−^* and *msn^−^* displayed FLs of 2.14±0.17 ns, 2.19±0.20 ns and 2.22±0.10 ns respectively. In all cases, stretching cells led to a variable additive activation [0.17 ns (*bsk^−^*), 0.13 ns (*hep^−^*), 0.11 ns (*slpr^−^*), and 0.07 ns (*msn^−^*), highest for *bsk^−^*] of the dJun-FRET biosensor when compared to resting dsRNA treated cells (Figure 1A to D and Tables S2 and S3). Comparing FL values for dsRNA treated cells under stretch to FL values of WT cells under stretch (WT^S)^, we observed that while the absence of Bsk results in a further response from the biosensor, *hep^−^, slpr^−^* and *msn^−^* cells showed less activity than the WT^S^ cells. Therefore, the presence of Hep, Slpr or Msn is necessary for the full activation of the biosensor in response to stretch, while Bsk seems to act independently in parallel restraining the activity/phosphorylation of the biosensor both in resting and stretch conditions. This confirmed a net negative input of Bsk on dJun phosphorylation in S2R+ cells, not only in resting conditions but also under stretch.

### Rl (ERK) but not P38s is an activator of dJun phosphorylation

ERKs and p38s have been implicated in the activation of AP1, and particularly Jun, in many processes, including stress responses. S2R+ cells were co-transfected with the dJun-FRET biosensor and dsRNAs for *rl*, *mpk2* (*p38α*) and *p38β* individually and FLIM values were measured at rest and in stretched conditions (2 hours).

Cells knocked down for *p38α* and *p38β* barely activated the dJun-FRET biosensor at rest (FL 2.38±0.16 ns for both *p38α^−^* and *p38β^−^* conditions) (Figure 1E and 1F). Under stretch, *p38α^−^* and *p38β^−^* cells display FL values of 2.23±0.12 ns and 2.31±0.17 ns respectively (Figure 1E and 1F). Similar to the JNK cascade elements loss of function conditions, these cells show an additive activation [0.15 ns (*p38α^−^*) and 0.07 ns (*p38β^−^*)] in the presence of mechanical stretch. If, as above, we compare FL values for *p38α^−^* and *p38β^−^* cells under stretch to FL values of WT cells under stretch (WT^S)^, they showed lower activity (Figure 1E and 1F). Hence, P38α and P38β are necessary for the full activation of the dJun-FRET biosensor in response to stretch.

In resting conditions, cells knocked down for *rl*, in contrast to JNK cascade elements and *p38s*, showed an increase in average FL value from 2.43±0.15 ns in WT^R^ cells to 2.52±0.15 ns (Figure 1G). Consequently, Rl, unlike Bsk, P38α or P38β, behaves as an activator of dJun phosphorylation at rest in S2R+ cells. In the presence of mechanical stretch *rl^−^* cells maintain a FL value of 2.52±0.15 ns (Figure 1G), indicating that in cells devoid of Rl, the dJun-FRET biosensor completely loses its ability to respond to the stretch activation. Thus, Rl is essential both for the activation of the dJun-FRET biosensor and for its response to stretch.

### Puc is an inhibitor of dJun phosphorylation

*puc* encodes for a well-characterized dual phosphatase that acts as a negative regulator of the JNK signaling pathway in many developmental processes in *Drosophila* [18]. S2R+ cells co-transfected with the dJun-FRET biosensor and dsRNA for *puc* showed an average FL value of 2.10±0.16 ns in resting conditions (Figure 1H). This FL value is lower than for WT^R^ cells (2.43±0.15 ns), indicating that Puc, as expected, inhibits the dJun-FRET biosensor phosphorylation. When *puc* ^−^ S2R+ cells were subjected to mechanical stretch they activated the dJun-FRET biosensor to the same extent as the WT^S^ cells with a FL value of 2.02±0.17 ns (Figure 1H). Thus, Puc is apparently not required for the dJun-FRET biosensor activation by mechanical stretch, even though it acts as an inhibitor of the phosphorylation of dJun in resting conditions.

### Double knockdowns at rest and in stretched conditions indicate complex epistatic interactions

To explore the epistatic relationships between Bsk, Rl and Puc in the response of the dJun-FRET biosensor, we interfered with their expression in pairs. The response of the dJun-FRET biosensor by FRET/FLIM was first analyzed in resting conditions (Table S1). P38s, which did not affect dJun-FRET biosensor activation were not included in this analysis.

**Figure 2:**
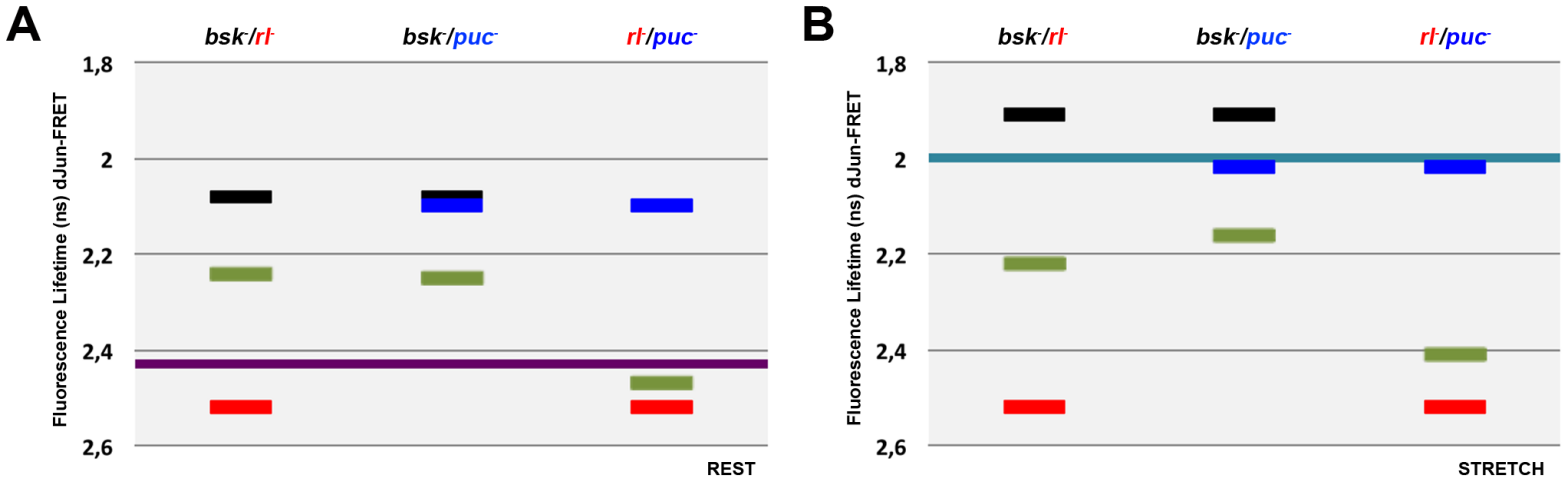
Epistatic interactions between *bsk, rl* and *puc* at rest and upon mechanical stretch. Graphical representation of the averaged FL values of cells co-transfected with the pAct-dJun-FRET biosensor and single or double combinations of dsRNAs. **A**) at rest. **B**) upon stretch. Black - *bsk^−^*; Red - *rl^−^*; Blue - *puc^−^*; pale Green - double treatment for each paired combination. Purple and Cyan bars represents the average FL for wild type cells at rest and upon stretch as in Figure 1.

When comparing to WT^R^ control cells, single knockdowns of *bsk* and *puc* resulted in different increments in the phosphorylation of the dJun-FRET biosensor at rest, while reduction in the levels of Rl decreased/inhibited its phosphorylation. Double knockdown of these regulators gave rise to a complex set of results. Inhibition of Bsk and Rl resulted in a moderate intermediate activation of the biosensor. On the contrary, the double knockdown of *bsk* and *puc* resulted in a moderate level of biosensor activation, much lower than that reached in single knockdowns for any of them. Finally, *puc* loss of function was tested in double knockdowns with *rl*. *puc* and *rl* double mutant cells showed a level of activation of the biosensor close to that observed in *rl* ^−^ cells (Figure 2A). In summary, at rest, Rl is epistatic over Puc and Bsk and Puc cancel each other; furthermore, the opposite activities of Bsk and Rl appear to be independent.

Under stretch, the activity of the dJun-FRET biosensor (WT^S^) was much higher than at rest (WT^R^), *bsk* knockdown induced a further activation of the biosensor, *puc* did not affect the dJun–FRET (WT^S^) biosensor activity and a single knockdown for *rl* blocked the stimulation of the biosensor by stretch. This scenario is remarkably different than that observed at rest anticipating specific changes in epistatic relationships. Double knockdowns of *bsk* and *rl* resulted, as at rest, in a moderate intermediate activation of the biosensor when compared to WT^S^. The double inhibition of Bsk and Puc resulted in a moderate level of biosensor activation compared to WT^S^, much lower than that reached in response to single knockdowns for any of them. Finally, under stretch, in *puc* double knockdown with *rl,* the activity of the biosensor was brought back to WT^R^ levels. The absence of Rl (an activator of the biosensor in single knockdowns) is epistatic to the stretch response preventing dJun phosphorylation (Figure 2B). In summary, after stretch some epistatic relationships defined at rest are conserved, Bsk and Puc cancel each other and the opposite activities of Bsk and Rl are independent. However, a new interaction was observed, where Rl and Puc activities became independent, although they are in some manner coordinated, being both necessary for the stretch response.

### Rho GTPases have distinct roles in the mechanical stretch activation of dJun

**Figure 3:**
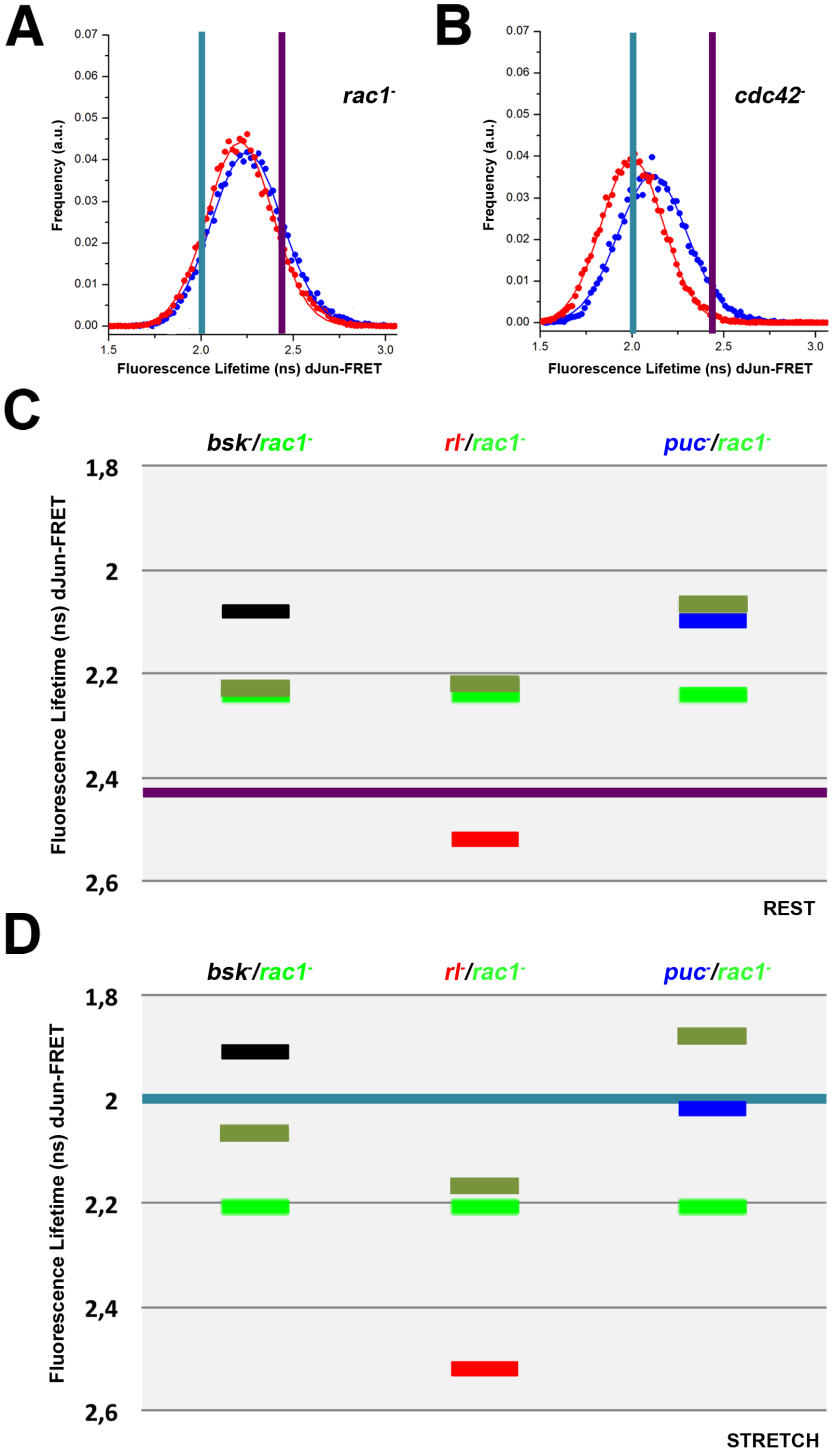
Rho GTPases have distinct roles during mechanical stretch activation; Epistatic relationships of *rac1* over *bsk, rl* and *puc*. *Drosophila* S2R+ cells were co-transfected with the pAct-dJun-FRET biosensor and different dsRNAs. **A)** *rac1^−^*. **B)** *cdc42^−^*. Fluorescence lifetimes (FL) for the donor mCFP were collected and curves representing data recorded from ∼75 cells for each condition are displayed. Blue and red data points denote the measurements obtained at rest or upon stretch as in Figure 1. Graphical representation of the averaged FL values of cells co-transfected with the pAct-dJun-FRET biosensor and single or double combinations of dsRNAs. **C)** at rest. **D)** upon stretch. Black - *bsk^−^*; Red - *rl^−^*; Blue - *puc^−^*; bright Green - *rac1^−^*; pale Green - double treatment for each pairwise combination. Purple and Cyan bars represents the average FL for wild type cells at rest and upon stretch as in Figure 1.

*Rac1* and *cdc42* are genes coding for Rho GTPases known to regulate the activity of the JNK pathway [23]. S2R+ cells at rest, treated with dsRNA for *rac1* and *cdc42* show a reduction (compared to WT^R^ cells) in the FL of the dJun-FRET biosensor (2.24±0.19 ns and 2.11±0.18 ns respectively) (Figure 3A and 3B and Tables S1 to S3). Rac1 and Cdc42, as is the case for the JNK cascade elements, inhibit the phosphorylation of dJun. In the presence of stretch, we observed as well an additive activation of the biosensor, 0.11 ns, for *cdc42^−^* cells (2.00±0.15 ns) but no differences for *rac1^−^* cells (0.03 ns) (2.21±0.18 ns) (Figure 3A and 3B and Tables S1 to S3). Thus, Rac1 activity is apparently epistatic over mechanical stretch and in the absence of Rac1, the FL values of resting and mechanically stretched cells are essentially the same. When compared to WT^S^, *cdc42^−^* stretched cells showed the same level of dJun-FRET biosensor activation suggesting that Cdc42 has no role in the dJun-FRET biosensor activation in response to stretch.

Considering this essential role of Rac1 for the stress response of S2R+ cells, we analyzed double knockdowns of *rac1* with *rl*, *bsk* and *puc*. Inhibition of Bsk and Rac1 in resting conditions led to an activation of the biosensor almost identical to that observed in *rac1^−^* cells, suggesting that in the absence of Rac1, the presence or absence of Bsk is irrelevant. *rac1* was also tested in double knockdowns with *rl* and *puc*. Remarkably, while Rac1 was epistatic over Rl (as it was for Bsk), Puc was epistatic over Rac1 (Figure 3C and Tables S1 and S3).

On the contrary to what happens at rest, in mechanically stretched cells the inhibition of Bsk and Rac1 resulted in intermediate biosensor activation and Rac1 was no longer epistatic. Rac1 was also tested with Rl and Puc. As at rest, Rac1 was epistatic to Rl. However, although the absence of Puc alone did not affect the level of biosensor activation in stretched cells, when combined with the loss of Rac1 expression, it resulted in hyperphosphorylation of the sensor. Rac1 and Puc thus exhibit synergic activities on stretched cells (Figure 3D and Tables S2 and S3). In summary, after stretch the epistatic relationships defined at rest were sharply altered.

### MAPKs Network Equilibrium Model

From the quantitative values obtained from the loss of function and epistatic experiments we reconstructed the network topology describing the interaction vectors amongst different modules, both at rest and after mechanical stretch. Importantly, in the experimental conditions applied, the level of activation of the dJun-FRET biosensor was at a steady state with neither oscillating behaviors nor bi-stable responses. Hence, considering the system at equilibrium it was possible to develop a mathematical formalism of enzymatic reaction systems. The network is described by non-linear equations, which relate the activity of the dJun-FRET biosensor to the activities and reaction coefficients of the components of the network and their upstream entries [24] (see Methods S1).

**Figure 4:**
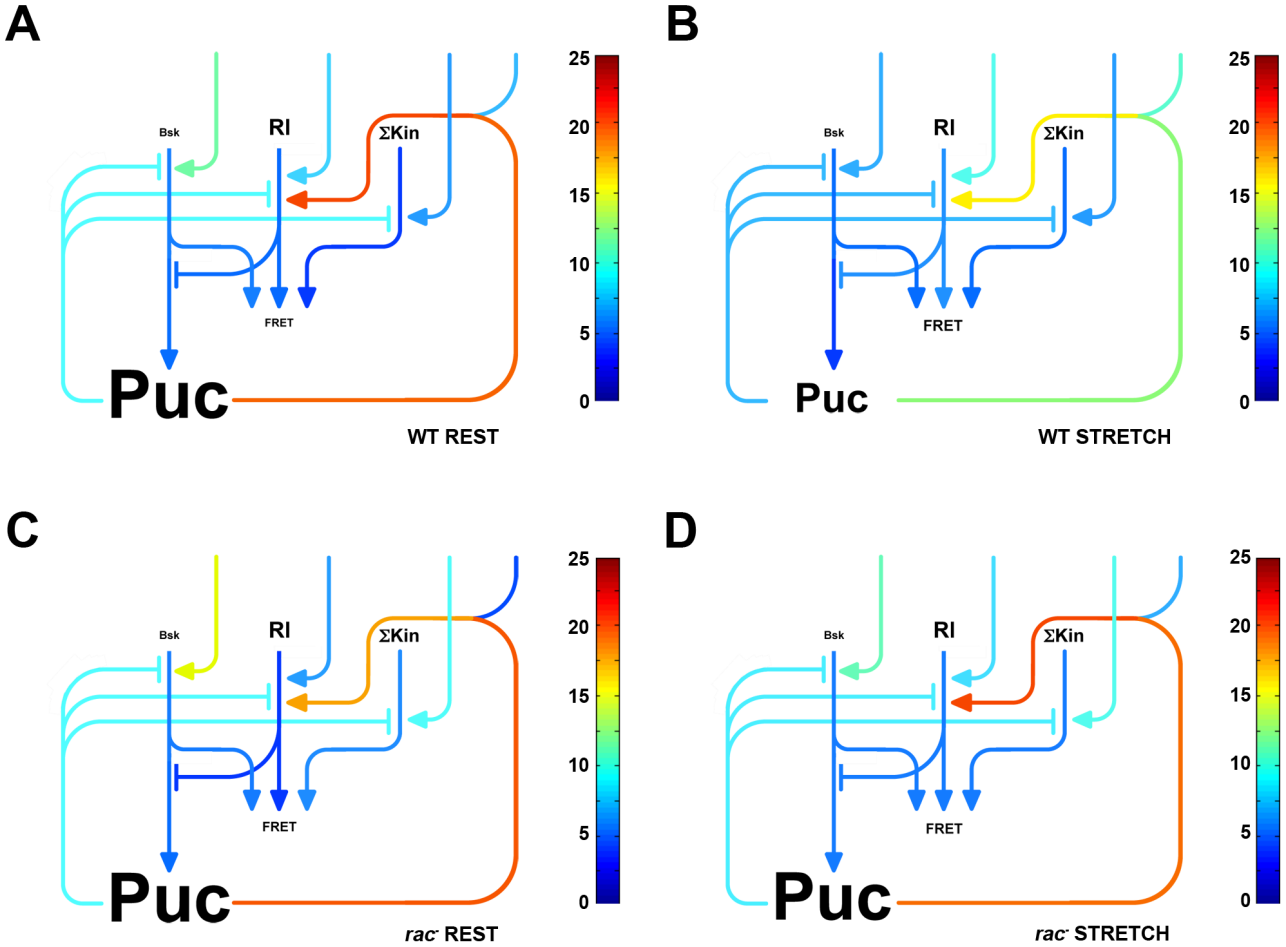
MAPKs Network Dynamic Model. To build the network, we took into account three elements, Puc, Bsk, and Rl plus an additional component (ΣKin) integrating all other potentially involved kinases (such as P38s). We considered that the dJun-FRET biosensor could be activated at different levels by Bsk, Rl and ΣKin, that the expression of Puc in response to dJun phosphorylation was only triggered by Bsk, and that Puc inhibit all kinases Bsk, Rl and ΣKin with different affinities. We further established two other biochemical links: a negative input from Rl onto Bsk function (activation of Puc expression) and a positive feedback loop from Puc upstream of Rl. We then determined a set of parameters allowing calculated activation ratios to best fit the FRET measurements of single, and double knockdowns and the control condition at rest (**A**) or upon stretch (**B**). We further evaluated the model taking into consideration the epistatic analysis performed on *rac1* at rest (**C**) and upon stretch (**D**). Components concentrations (font size) and levels of activation or repression (rainbow look up table) are displayed according to logarithmic scales following the values defined in Table S4.

**Figure 5:**
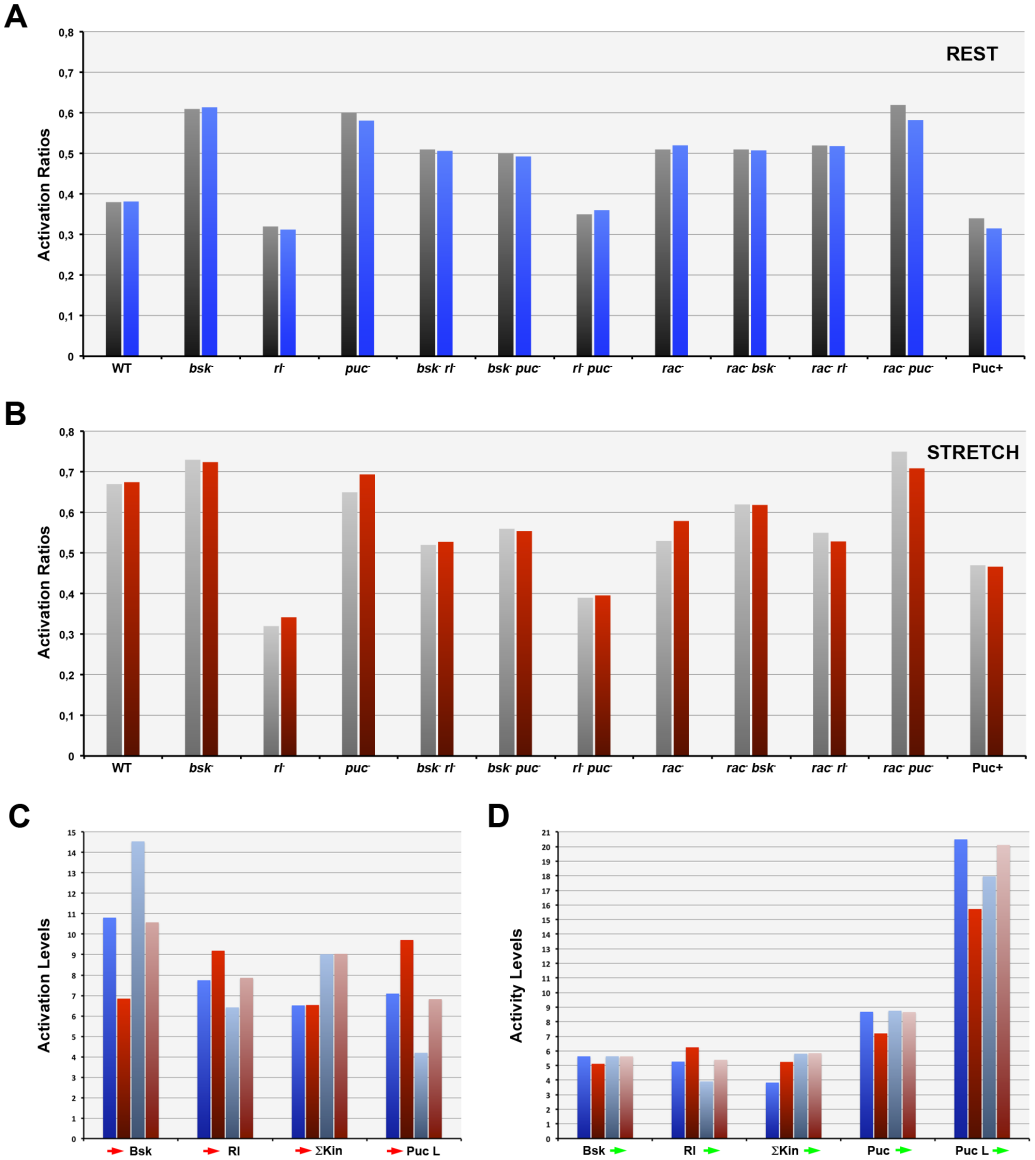
Mechanical stretch and the absence of Rac1 alter the magnitude of extrinsic inputs and intrinsic interactions within the MAPK network. The different experimental conditions and control experiments yielded specific FL values from the dJun-FRET biosensor FLIM measurements. Fitted AR measurements applying the network model very precisely reproduced the experimental data (AR) for all conditions at rest and upon stretch. A) Dark grey – Experimental AR at rest; Dark blue – Fitted AR at rest. B) Pale grey – Experimental AR upon stretch; Dark red – Fitted AR upon stretch. C) The extrinsic inputs into the network (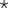
Bsk, 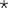
Rl; 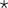
ΣKin; 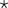
Puc loop) show different activation levels at rest (dark blue) and upon stretch (dark red) except for ΣKin. When *rac1* expression is abolished, these values are altered but their ratio is sustained (pale blue at rest; pale red upon stretch). See also Figure 4. D) The intrinsic positive and negative interactions (activity levels) between the network different nodes (Bsk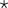, Rl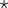; ΣKin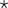; Puc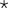; Puc loop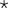) are distinctively modified both upon stretch and, synergically, in the absence of Rac1. Components concentrations and levels of activation are displayed as in Figure 4.

To build the network, we considered three main elements, Puc, Bsk, and Rl in addition to a fourth component (ΣKin) that integrates all other extrinsic inputs that potentially affect the biosensor (such as P38s and/or other kinases). To start, we imposed certain basic assumptions [15], [18], [25]: the dJun-FRET biosensor could be activated at different levels by Bsk, Rl and ΣKin, the expression of Puc in response to dJun phosphorylation was triggered by Bsk only, and Puc could inhibit all kinases Bsk, Rl and SKin albeit with different affinities. We further implemented two other biochemical links: a negative input from Rl onto Bsk function (activation of Puc expression) and a positive feedback loop from Puc upstream of Rl (see Discussion). The ultimate network model considers all these associations. It considers seven independent parameters [the concentrations of Bsk (A1), Rl (A2) and ΣKin (A3) and their activation by external inputs (ω_1_, ω_2_, and ω_3_ respectively) plus the external input on the hypothesized Puc-mediated positive feedback loop on Rl activity (β)] plus two *ad hoc* cooperativity exponents (two power terms applied to Bsk activity introducing cooperativity, and to the exponential amplification of the input of Puc in Rl activity). Applying a Monte Carlo algorithm, and modulating these parameters and cooperative exponents we calculated activation ratios (AR) to best fit the FRET measurements of single, double knockdowns and wild type cells at rest (**RWT**). To simplify the fitting, we a-dimensioned the model by setting the ΣKin concentration (A3) to a constant value. Further, we reached the most accurate approximations when setting the power terms to 2 (Bsk activity) and 5 (Puc input in Rl) respectively. In this way, we attained a precise quantitative fit of the FRET values for all experimental single and double knockdowns (Figure 4A and 5A, Table S4 and Methods S1).

The model was re-tested for the stretch conditions (**SWT**). Importantly, we found that, upon stretch, a 28X increase in the upstream activation of Rl (ω_2_) along with a 420X increase in the extrinsic input to the Puc positive loop to Rl (β) and a 9200X decrease in the upstream activation of Bsk (ω_1_) without modifying the network topology, precisely fits the decrease on the FL (2.43 to 2.0 ns) of the dJun-FRET biosensor (**RWT** vs. **SWT**) (Compare Figures 4A and 4B and see Figures 5A to 5C). These changes in external inputs, upon mechanical stimulation of S2R+ cells, result within the network and according to the model in reductions of Bsk activity (3.2X), the inhibitory activity of Puc (30X), and the net activity of the Puc positive loop on Rl (59000X) and in increments in the activities of Rl (10X) and ΣKin (26X) (**RWT** vs. **SWT**) (Figure 5D).

We further refined the model taking into consideration the epistatic analysis performed on *rac1* at rest (**RRac1**) and under stretch (**SRac1**) (compare Figures 4C and 4D). The absence of Rac1 results in an enhanced activation of Bsk (ω_1_ - 5400X) and SKin (ω_3_ - 320X), a reduced activation of Rl (ω_2_ - 21X) and the extrinsic activation of the Puc to Rl positive loop (β - 790X) when comparing to the WT condition. Importantly, these changes take place irrespective of the cells' biomechanical condition (**RWT** vs. **RRac1** and **SWT** vs. **SRac1**) (Figure 5C) indicating that Rac1 does not influence the impact of mechanical stress in the system (all network inputs). On the other hand, within the core of the network, Rac1 depletion at rest (**RWT** vs. **RRac1**) results in an increment of SKin activity (95X) and reductions of Rl (30X) and the net Puc positive loop on Rl (140X) activities, while the activities of Bsk and Puc are unaffected (Figure 5D). Upon stretch, the absence of Rac1 (**SWT** vs. **SRac1**) results in the increment in activities of Bsk (3,2X), ΣKin (4X), Puc (29X) and the net Puc positive loop on Rl (24000X) while Rl activity decreases (7X) (Figure 5D). Remarkably, in the absence of Rac1 (**RRac1** vs. **SRac1**), cell stretching results in an increase of Rl activity (30X) and the net Puc positive loop on Rl (140X), barely affecting Bsk, ΣKin and Puc activities (Table S4 and Figure 5D).

### Gain of Function Analysis

The overexpression of Puc resulted in a mild inhibition of the biosensor phosphorylation at rest (2.49±0.14 ns) (**RPuc+**). Upon stretch, this overexpression resulted again in a partial inhibition of the biosensor activity (2.30±0.14 ns) (**SPuc+**) (Table S4). In both cases, as expected, the biosensor response was opposite to that observed in *puc* loss of function conditions.

**Figure 6:**
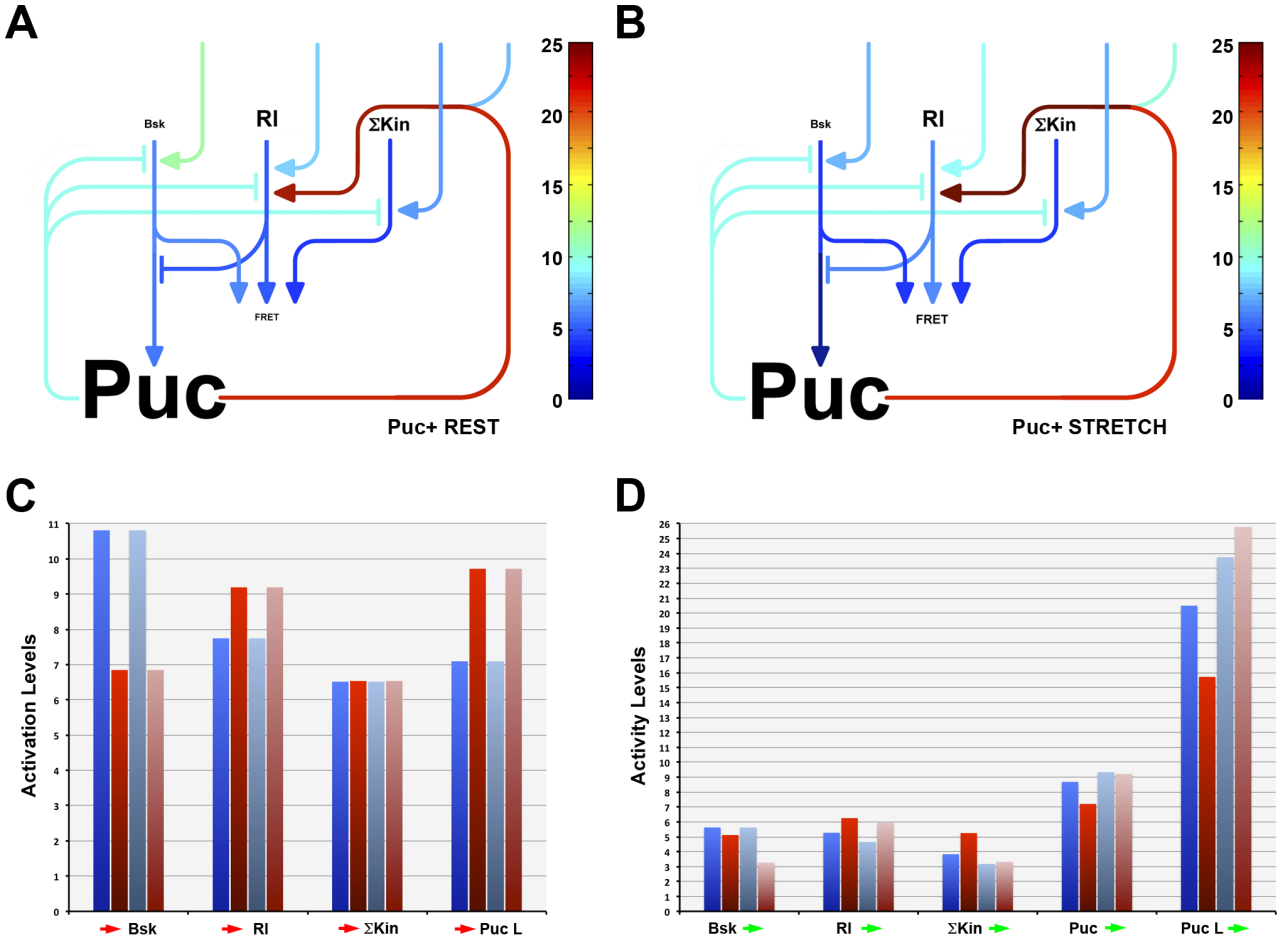
*puc* gain of function does not affect the MAPK network topology but influences intrinsic network interactions. We calculated activation ratios to best fit the FRET measurements upon Puc overexpression at rest (**A**) or upon stretch (**B**). The extrinsic inputs into the network (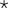
Bsk, 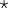
Rl; 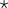
ΣKin; 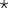
Puc loop) (**C**) and the intrinsic positive and negative interactions (activity levels) between the network's different nodes (Bsk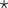, Rl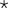; ΣKin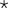; Puc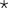; Puc loop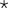) (**D**) were determined by fitting. Components concentrations and levels of activation or repression are displayed as in Figure 4.

Our model predicts that an increase of Puc levels should not affect the extrinsic inputs to the network (ω_1_, ω_2_, ω_3_ and β) and indeed this is the case. On the other hand, the modeled Puc overexpression points to an increase in Puc levels and increments of different magnitudes in the Puc inhibitory capacity and the net Puc positive loop on Rl activity both at rest and after stretching [4,5X and 1800X (**RWT** vs. **RPuc+**) vs. 100X and 1,1×10^10^X (**SWT** vs. **SPuc+**) respectively]. Further, Puc overexpression, when compared to the WT condition, does not affect Bsk activity at rest, but promotes its decrease 70X upon stretch. Meanwhile, Rl and Σkin activities are both reduced 4X at rest and decreased upon stretch 2X and 85X respectively (Figure 6).

Summarizing, none of the interactions between the different elements or the network topology are modified in response to Puc overexpression. A simple quantitative modulation of the intrinsic interactions between the network nodes fit the model parameters to the experimental FL values both before and after mechanical stretch.

## Discussion

An extensive literature supports that MAPK pathways activities are linked by undefined mechanisms facilitating their crosstalk [21]. By resorting to the activity of a dJun-FRET biosensor in *Drosophila* S2R+ cells in culture [15] we propose a functional network model linking individual MAPK cascades at rest or in the presence of mechanical stretch.

Surprisingly, we found that knocking down different elements of the JNK cascade resulted in an increase in the phosphorylation of the dJun-FRET biosensor in either condition, while inactivating the inhibitory dual-specificity MAPK phosphatase Puc also led to its activation. This drew a distinction with the observed biosensor inhibition consequence of knocking down Rl, an ERK homologue. The apparent contradiction between the known direct activation of dJun by Bsk and the activation of the biosensor after knocking down *bsk* and other members of the JNK cascade was solved by generating a network model taking into account cross-regulatory links between the JNK and ERK pathways.

To generate a MAPK network model by non-linear equations we considered a set of different literature supported evidences. First, the AP1 complex, mediating the transcription of *puc* [18], is composed of Jun and Fos, both of them being phosphorylated by Bsk. However, mammalian ERK can also phosphorylate Fos, albeit on distinct residues, resulting in the transcriptional regulation of different target genes by the AP1 complex [26]. This suggests that in S2R+ cells Rl may act as a repressor of the JNK mediated expression of *puc*. Second, the Puc dual-specificity phosphatase, which mainly operates on the phosphorylated form of Bsk can also impinge on ERK (Rl) signaling [25] and, potentially, on other kinases. Finally, as stated above, *bsk* and *puc* knockdowns increase the FRET signal/activation of the dJun-FRET biosensor, suggesting that both proteins behave as effective inhibitors. However, previous work has shown that Bsk is a direct activator of dJun driving the expression of Puc, which feeds back negatively to the activity of JNK. Considering the results of their single knockdowns one would assume that the double knockdown of these genes should activate the biosensor even more. However, this is not the case, implying the existence of a positive feedback loop from Puc upstream of the MAPKs. Indeed, it has been shown that SEK1, a kinase upstream of MAPKs is negatively regulated by phosphorylation [27] and it has been further reported that JNK is indirectly activated by JKAP, a dual-specificity phosphatase, and by its human orthologue JSP1 [28]. Thus, a positive loop from Puc impacting on Rl activity might be potentially feasible.

The model we developed indicates that the effective inhibition of the dJun-FRET biosensor by Bsk does not imply different affinities of the proteins involved or a specific change of the network topology in S2R+ cells. A negative regulation of MAPK activities by Puc, a negative input of Rl on Bsk activity, and an activation of the ERK pathway by Puc are sufficient to account for all the experimental measurements of the biosensor activity. This holds good both at rest and upon stretch. The model also denotes that in S2R+ cells the concentration of Rl is 4X higher than Bsk (and these are not altered upon stretch). Further, Puc concentration is 1000X that of Bsk at rest, and drops 30X upon stretch thus reducing its influence on the activities of Bsk, Rl and Σkin.

The implication of Rac1 in the response to mechanical stress in multiple cell lines has been thoroughly sustained [29], [30]. However, we found that the level of activation of the biosensor is not affected by the cells biomechanical condition (at rest or upon stretch) when Rac1 is inhibited in S2R+ cells (Figure 3A). Importantly, our model indicates that the extrinsic inputs to the network (ω_1_, ω_2_, ω_3_ and β) are, once Rac1 expression is inhibited, much bigger for ω_1_ and ω_3_ and smaller for ω_2_ and β) than for WT cells. However, they are modulated in the same proportions between cells at rest and under stretch irrespectively of the presence of Rac1. Still, without Rac1, the intrinsic interactions between the different nodes display disparate responses than WT cells. In particular, the activities of Bsk, Σkin and Puc, which are different between cells at rest and upon stretch in the WT condition, are essentially locked at a particular level in the absence of Rac1. In contrast, Rl activity is different in cells at rest and under strech when Rac1 is not present and decreases differently at rest (22X) and upon stretch (7X) when compared to WT cells. This emphasizes the key role of Rl modulating the level of activation of the biosensor (Figure 1G).

In the proposed model, the activation of the dJun-FRET biosensor varies within a specific dynamic range in response to the concerted actions of multiple negative and positive loops. It is intriguing to find that in comparing the different experimental conditions assesed some kinases duplicate or triplicate their activity, while others change their levels of activity up to 5 orders of magnitude. Although at the origin of these differences we could place the disparity between the fine-tuning of activity levels vs the activation from a negligible ground state, systems-level precise behavioral regulation may also be very influencial. Thus, global effects such as competition for substrates, multisite phosphorylation and kinetic proofreading regulating specificity by phosphatases in complex mixtures of proteins [31] can account for dramatic differences in individual network-elements activities. Signaling cascades can transduce information in different ways [32]. Cascades may behave gradually when the activity of the terminal kinase quantitatively reflect the concentration of the extracellular stimulus. Alternatively, the cascade may act as an ultrasensitive switch that responds in a all-or-none manner: amplification and cellular commitment only occur once a threshold stimulus is reached. Theoretical studies revealed that minimal models of multi-step protein kinase cascades show gradual dose-response behavior at steady state [33]. Indeed, the intrinsic hierarchical nature of MAPK pathways cascades prompts to major signal amplification outcomes. A classical example is the neurite outgrowth induced by NGF vs the proliferative signal without neurite formation promoted by EGF in PC12 cells via the same signal transduction MAPK cascade. These differential responses are thought to be determined by the duration of MAPK activation; NGF induces sustained MAPK activation for several hours and translocation to the nuclei, but EGF leads to short-lived activation [34].

The model also explains how crosstalk within pathways can integrate responses differing markedly between cells at rest and under mechanical stress. Thus, may be useful in the understanding of how mechanical (or eventually chemical or hormonal) inputs may disturb signal processing. This is particularly important in the context of cancer and tumor related conditions such as hypoxia, as many cancer cells and cells exposed to low oxygen levels display increased expression of dual specificity phosphatases [35]. This network model may provide possible explanations for the complex behavior of MAPK systems in different oncogenic paradigms resorting to MAPKs hyperactivity and it may help clarify the regulatory mechanisms linked to the transitions from a normal apoptotic cell to uncontrolled proliferation [14], [36].

This canonical model forms a basis for experimental design and can be tailored to different experimental systems on two levels, by parameter estimation and by extending the model to incorporate different MAPK isoforms and upstream, downstream and structural elements. Such refined models possess quantitative predictive power and cannot only be used for identifying gaps in knowledge, but also for elucidating the effect of drugs, thus building the theoretical basis for identifying optimal treatment strategies.

## Materials and Methods

### Cell culture

*Drosophila* S2R+ cells were grown in Schneider's *Drosophila* medium (GIBCO, Invitrogen) supplemented with 10% heat-inactivated fetal bovine serum (GIBCO, Invitrogen) at 25°C. Penicillin and streptomycin were included at 100 units/ml and 100 µg/ml, respectively.

### RNAi and overexpression treatments

200,000 cells were seeded in a 24 well plate and incubated at 25°C overnight. Cells were co-transfected with different dsRNAs (∼5 ug of RNAi in each reaction) or ∼5 µg of a *puc* overexpression construct (pAct 5C-Puc) and 2 µg/µl pAct-dJun-FRET biosensor simultaneously, at ∼80% confluence using Effectene (Qiagen) following the manufacturer's instructions. Transfected cells were incubated for 4 days and then re-plated on collagen-coated silicone membranes in medium deprived of serum, one day prior to vacuum-assisted stretch FLIM analysis. The dsRNAs and information about potential off-targets were obtained from the DRSC (http://flyrnai.org). Cells transfected with dsRNAs were re-plated on collagen-coated silicone membranes, in medium deprived of serum, one day prior to vacuum-assisted stretch FLIM analysis.

### Modeling

To model the interaction network leading to the activation of the dJun-FRET biosensor in resting and stretch conditions we applied a system of non-linear equations. Details are presented in the Methods S1.

## Supporting Information

Table S1 **Fluorescence Lifetimes (FL) of S2R+ cells subjected to distinct single and double knockdowns at rest.** FL measurements not significantly differing of the wild type (WT) values are displayed in blue. FL values significantly smaller than WT ones are displayed in red. FL values significantly bigger than WT ones are displayed in green.
(PDF)

Table S2 **Fluorescence Lifetimes (FL) of S2R+ cells subjected to distinct single and double knockdowns upon stretch.** FL measurements not significantly differing of the wild type (WT) values are displayed in blue. FL values significantly smaller than WT ones are displayed in red. FL values significantly bigger than WT ones are displayed in green.
(PDF)

Table S3 **Differences of Fluorescence Lifetimes (FL) at rest vs stretch conditions of S2R+ cells subjected to distinct single and double knockdowns.** FL measurements not significantly differing between both conditions are displayed in blue. FL values significantly smaller upon stretch versus resting conditions are displayed in red.
(PDF)

Table S4 **Consolidated parametric fitted values for the MAPK Network for the distinct single and double knockdowns and the overexpression of Puc at rest and upon stretch.** Experimental AR (FL), fitted AR, A1 (**[Bsk]**), A2 (**[Rl]**), A3 **([Σkin]**), Puc (**[Puc]** and **<Puc**), Omega1 (**>Bsk Ext**), Omega2 (**>Rl Ext**), Omega3 (**>Σkin Ext**), Beta (**>Puc L Ext**), K1 (**<Bsk**), K2 (**<Rl**), K3 (**<Σkin**), K1∧2.[(1-K2)/(1+K2)] [**>Puc <(Bsk.Rl)**], Puc∧5 (**<Puc L**) and Beta.Puc∧5 (**<Puc L>Puc L Ext**) values for each experimentally analyzed condition at rest and upon stretch. Shadowed in green are the values represented in Figures 4A to 4D, Figures 5C and 5D and Figures 6A to 6D. Shadowed in Orange are the values presented in Figure 5A and 5B.
(PDF)

Methods S1 **Supporting Methods.**
(PDF)
