# Supplementary material for: Plasticity of the MAPK Signaling Network in Response to Mechanical Stress
Source: PLoS One. 2014 Jul 15;9(7):e101963. doi: 10.1371/journal.pone.0101963 (PMC4099004; doi:10.1371/journal.pone.0101963)
Supplement: Table S1 — Fluorescence Lifetimes (FL) of S2R+ cells subjected to distinct single and double knockdowns at rest. FL measurements not significantly differing of the wild type (WT) values are displayed in blue. FL values significantly smaller than WT ones are displayed in red. FL values significantly bigger than WT ones are displayed in green. (PDF) [file pone.0101963.s001.pdf]

Table S1

	WT	*msn*	*slpr*	*hep*	*bsk*	*rl*	*puc*	*rac1*	*p38a*	*p38b*	*cdc42*
WT	2.43										
msn		2.27									
slpr			2.30								
hep				2.27							
bsk					2.08	2.24	2.25	2.23			
rl						2.52	2.47	2.22			
puc							2.10	2.07			
rac1								2.24			
p38a									2.38		
p38b										2.38	
cdc42											2.11

Table S1. Fluorescence Lifetimes (FL) of S2R+ cells subjected to distinct single and double knockdowns at rest

FL measurements not significantly differing of the wild type (WT) values are displayed in blue. FL values significantly smaller than WT ones are displayed in red. FL values significantly bigger than WT ones are displayed in green.
