# Supplementary material for: Plasticity of the MAPK Signaling Network in Response to Mechanical Stress
Source: PLoS One. 2014 Jul 15;9(7):e101963. doi: 10.1371/journal.pone.0101963 (PMC4099004; doi:10.1371/journal.pone.0101963)
Supplement: Table S3 — Differences of Fluorescence Lifetimes (FL) at rest vs stretch conditions of S2R+ cells subjected to distinct single and double knockdowns. FL measurements not significantly differing between both conditions are displayed in blue. FL values significantly smaller upon stretch versus resting conditions are displayed in red. (PDF) [file pone.0101963.s003.pdf]

Table S3

	WT	*msn*	*slpr*	*hep*	*bsk*	*rl*	*puc*	*rac1*	*p38a*	*p38b*	*cdc42*
WT	-43										
msn		-7									
slpr			-11								
hep				-13							
bsk					-17	-2	-9	-16			
rl						0	-6	-5			
puc							-8	-19			
rac1								-3			
p38a									-15		
p38b										-7	
cdc42											-11

Table S3. Differences of Fluorescence Lifetimes (FL) at rest vs stretch conditions of S2R+ cells subjected to distinct single and double knockdowns

FL measurements not significantly differing between both conditions are displayed in blue. FL values significantly smaller upon stretch versus resting conditions are displayed in red.
