# Supplementary material for: Plasticity of the MAPK Signaling Network in Response to Mechanical Stress
Source: PLoS One. 2014 Jul 15;9(7):e101963. doi: 10.1371/journal.pone.0101963 (PMC4099004; doi:10.1371/journal.pone.0101963)
Supplement: Table S4 — Consolidated parametric fitted values for the MAPK Network for the distinct single and double knockdowns and the overexpression of Puc at rest and upon stretch. Experimental AR (FL), fitted AR, A1 ([Bsk]), A2 ([Rl]), A3 ([Σkin]), Puc ([Puc] and <Puc), Omega1 (>Bsk Ext), Omega2 (>Rl Ext), Omega3 (>Σkin Ext), Beta (>Puc L Ext), K1 (<Bsk), K2 (<Rl), K3 (<Σkin), K1∧2.[(1-K2)/(1+K2)] [>Puc <(Bsk.Rl)], Puc∧5 (<Puc L) and Beta.Puc∧5 (Puc L Ext) values for each experimentally analyzed condition at rest and upon stretch. Shadowed in green are the values represented in Figures 4A to 4D, Figures 5C and 5D and Figures 6A to 6D. Shadowed in Orange are the values presented in Figure 5A and 5B. (PDF) [file pone.0101963.s004.pdf]

Table S4

		EXPERIMENTAL FRET	CALCULATED FRET	[Bsk]	[Rl]	[Σkin]	[Puc]	>Bsk Ext	>Rl Ext	>Σkin Ext	>Puc L Ext	<Bsk	<Rl	<Σkin	>Puc <(Bsk.Rl)	<Puc	<Puc L	<Puc L . >Puc L Ext
		EXPERIMENTAL FRET	CALCULATED FRET	A1	A2	A3	Puc	Omega1	Omega2	Omega3	Beta	K1	K2	K3	K1^2 . [(1-K2) / (1+K2)]	Puc	Puc^5	Beta.Puc^5
WT	Resting	0,38	0,38165	0,42781	1,785	1	479,07	64365	55,652	3,2672	12,37	0,42465	0,18578	0,0067736	0,152075109	479,07	2,52345E+13	3,12151E+14
	Stretched	0,67	0,67482	0,42781	1,785	1	15,897	7,0211	1553,4	3,4246	5214,2	0,13106	1,7669	0,17724	0,006207931	15,897	1015256,716	5293751570
Bsk -	Resting	0,61	0,61417	0,016196	1,785	1	0,56164	64365	55,652	3,2672	12,37	0,016196	0,72232	0,85331	0,000152301	0,56164	0,055884343	0,691289329
	Stretched	0,73	0,72426	0,016196	1,785	1	0,32775	7,0211	1553,4	3,4246	5214,2	0,015473	1,6985	0,91265	8,8721E-05	0,32775	0,00378193	19,71974011
Rl -	Resting	0,32	0,31228	0,42781	0,26097	1	549,72	64365	55,652	3,2672	12,37	0,42419	0,023991	0,0059083	0,175721423	549,72	5,02005E+13	6,2098E+14
	Stretched	0,32	0,34186	0,42781	0,26097	1	19,586	7,0211	1553,4	3,4246	5214,2	0,11289	0,25772	0,14882	0,010132742	19,586	2882230,774	15028527700
Puc -	Resting	0,6	0,5811	0,42781	1,785	1	54,353	64365	55,652	3,2672	12,37	0,42745	0,90305	0,056702	0,096010879	54,353	474370419	5867962083
	Stretched	0,65	0,69413	0,42781	1,785	1	8,1548	7,0211	1553,4	3,4246	5214,2	0,19793	1,7757	0,29575	0,01411402	8,1548	36063,39189	188041738
Bsk - Rl -	Resting	0,51	0,5064	0,016196	0,26097	1	0,80164	64365	55,652	3,2672	12,37	0,016196	0,20677	0,80298	0,000217366	0,80164	0,331052519	4,09511966
	Stretched	0,52	0,52765	0,016196	0,26097	1	0,6434	7,0211	1553,4	3,4246	5214,2	0,014836	0,26041	0,84184	0,000174631	0,6434	0,110256774	574,900873
Bsk - Puc -	Resting	0,5	0,49274	0,016196	1,785	1	0,16207	64365	55,652	3,2672	12,37	0,016196	0,0024585	0,95274	0,000261667	0,16207	0,000111818	0,001383193
	Stretched	0,56	0,55428	0,016196	1,785	1	0,12713	7,0211	1553,4	3,4246	5214,2	0,015908	0,26345	0,96421	0,000200296	0,12713	3,32078E-05	0,173152163
Puc - Rl -	Resting	0,35	0,36022	0,42781	0,26097	1	88,998	64365	55,652	3,2672	12,37	0,42722	0,1004	0,035411	0,165864166	88,998	5583432055	69067054518
	Stretched	0,39	0,39556	0,42781	0,26097	1	11,348	7,0211	1553,4	3,4246	5214,2	0,16352	0,25907	0,23182	0,021236937	11,348	188190,0405	981260508,9
Rac -	Resting	0,51	0,52014	0,42781	1,785	1	566,55	3,47E+08	2,6142	1042,6	0,015689	0,42781	0,0081988	0,64792	0,181533043	566,55	5,83702E+13	9,1577E+11
	Stretched	0,53	0,57899	0,42781	1,785	1	455,27	37866	72,969	1092,9	6,613	0,42273	0,24658	0,70592	0,143352735	455,27	1,95589E+13	1,29343E+14
Rac - Bsk -	Resting	0,51	0,50767	0,016196	1,785	1	0,95209	3,47E+08	2,6142	1042,6	0,015689	0,016196	0,015866	0,99909	0,000258214	0,95209	0,782330061	0,012273976
	Stretched	0,62	0,61874	0,016196	1,785	1	0,60182	37866	72,969	1092,9	6,613	0,016195	0,60725	0,99945	0,000163184	0,60182	0,078946537	0,522073446
Rac - Rl -	Resting	0,52	0,5182	0,42781	0,26097	1	570	3,47E+08	2,6142	1042,6	0,015689	0,42781	0,0011914	0,64654	0,182803604	570	6,01692E+13	9,43995E+11
	Stretched	0,55	0,52862	0,42781	0,26097	1	541,52	37866	72,969	1092,9	6,613	0,42178	0,030989	0,66867	0,17255118	541,52	4,65664E+13	3,07943E+14
Rac - Puc -	Resting	0,62	0,58238	0,42781	1,785	1	93,154	3,47E+08	2,6142	1042,6	0,015689	0,42781	0,048727	0,91798	0,174517673	93,154	7014674775	110053232,5
	Stretched	0,75	0,70883	0,42781	1,785	1	50,887	37866	72,969	1092,9	6,613	0,42724	1,0516	0,95551	0,088971543	50,887	341219813,2	2256486624
Puc +	Resting	0,34	0,31539	0,42781	1,785	1	2144,3	64365	55,652	3,2672	12,37	0,41402	0,045156	0,0015214	0,164006675	2144,3	4,53344E+16	5,60786E+17
	Stretched	0,47	0,46665	0,42781	1,785	1	1630	7,0211	1553,4	3,4246	5214,2	0,0018349	0,87102	0,0020965	1,79948E-06	1630	1,15064E+16	5,99965E+19

Table S4. Consolidated parametric fitted values for the MAPK Network for the distinct single and double knockdowns and the overexpression of Puc at rest and upon stretch

Experimental AR (FL), fitted AR, A1 (**[Bsk]**), A2 (**[Rl]**), A3 **([Σkin]**), Puc (**[Puc]** and **<Puc**), Omega1 (**>Bsk Ext**), Omega2 (>**Rl Ext**), Omega3 (>**Σkin Ext**), Beta (>**Puc L Ext**), K1 (<**Bsk**), K2 (<**Rl**), K3 (<**Σkin**), K1^2.[(1-K2)/(1+K2)] [>**Puc <(Bsk.Rl)**], Puc^5 (<**Puc L**) and Beta.Puc^5 (<**Puc L.>Puc L Ext**) values for each experimentally analyzed condition at rest and upon stretch. Shadowed in green are the values represented in Figures 4A to 4D, Figures 5C and 5D and Figures 6A to 6D. Shadowed in Orange are the values presented in Figure 5A and 5B.
