# Supplementary material for: Plasticity of the MAPK Signaling Network in Response to Mechanical Stress
Source: PLoS One. 2014 Jul 15;9(7):e101963. doi: 10.1371/journal.pone.0101963 (PMC4099004; doi:10.1371/journal.pone.0101963)
Supplement: Methods S1 — Supporting Methods. (PDF) [file pone.0101963.s005.pdf]

METHODS S1

Plasticity of the MAPK signaling network in response to mechanical stress

Andrea M. Pereira et al

dsRNAs

The following dsRNAs were employed:

dsRNA	Amplicons	Off targets
basket (bsk)	DRSC03499, DRSC36595	0, 0
hemipterous (hep)	DRSC20337	1
slipper (slpr)	DRSC23321	0
misshapen (msn)	DRSC25188	0
mpk2 (p38α)	DRSC16743	0
p38β	DRSC03586	0
rolled (rl)	DRSC21814	0
puckered (puc)	DRSC26308, DRSC36627	1, 0
cdc42	DRSC25134	0
rac1	DRSC08688, DRSC25131	2, 0
GFP	-	

Cell stretching: Stage Flexer

The custom-built Stage Flexer consists of a double ringed frame, with an inverted cup-like plastic structure that supports a matrix bonded silicone rubber membrane in a single 35 mm well. The culture membrane is fixed in position above the plastic support with the help of a rubber seal through a groove in between the two rings. The membrane can be deformed by suction from below by applying negative pressure through an inlet drilled in the lower ring. The amount of strain deformation applied to the flexible substrate was calibrated as described, and in our experiments was routinely set at 2.5 % uniform stretch. Cells growing on the deformable membrane are accordingly subjected to mechanical stress by centrifugal stretch. The silicone deformable membranes were coated with collagen.

FLIM experiments

Frequency-domain FLIM experiments on transiently transfected *Drosophila* cells were performed using a Nikon TE2000-U inverted wide-field microscope and a Lambert Instruments Fluorescence Attachment (LIFA; Lambert Instruments, Roden, The Netherlands) for lifetime imaging. A light-emitting diode (Lumiled LUXEON III, λ max = 443 nm) modulated at 40 MHz was used to excite mCFP. Fluorescence detection was performed by a combination of a modulated (40 MHz) image intensifier (II18MD; Lambert Instruments) and a CCD camera (CCD-1300QD; VDS Vosskühler, Osnabrück, Germany) used at 2×2 binning (640×512 pixels). The emission of mCFP was detected

through a narrow emission filter (475/20 nm; Semrock, Rochester, U.S.A.) to suppress any crosstalk from mYFP fluorescence emission. FLIM measurements were calibrated with a 50 µM solution of pyranine (HPTS), the lifetime of which was set to 5.4 ns. All FLIM images were calculated from phase stacks of 12 recorded images, with exposure times of individual images of *Drosophila* cells ranging from 200 to 400 ms. Fluorescence lifetimes were calculated from several regions of interest (ROIs) comprising individual cells. Approximately 75 cells (ROIs) were selected for each condition. The obtained FL (pixel) values determined for each individual cell were summed to get FL histograms. These were fitted to Gaussian functions by using the OriginLab 6.0 software, from which the centers of the distributions and the distribution widths were extracted (the FL errors reported (±) are half the distribution width of the FL histograms). The experiments were performed at least three times and the data integrated into the histograms.

Experimental Data Conversion

The single and double RNAi knockdowns and control experiments at rest and under stretch conditions yielded precise FLIM dJun-FRET biosensor measurements. These data (FL) were converted into activation ratio (AR) values assuming a linear relationship. The maximum FL measured, corresponding to the minimum AR, is smaller than 3 ns, while the minimum FL was bigger than 1.5 ns. We used these values as 0 and 1 ARs respectively, so that

$$AR = (3 - FL)/1.5$$

	**FL**		**AR**	
	**Rest**	**Stretch**	**Rest**	**Stretch**
**WT**	2,43	2,00	0,380	0,667
bsk$^-$	2,08	1,91	0,613	0,727
rl$^-$	2,52	2,52	0,320	0,32
puc$^-$	2,10	2,02	0,600	0,653
bsk$^-$ **+ *rl***$^-$	2,24	2,22	0,507	0,52

bsk^{-} + ***puc***$^{-}$	2,25	2,16	0,500	0,56
rl^{-} + ***puc***$^{-}$	2,47	2,41	0,353	0,393
rac1^{-}	2,24	2,21	0,507	0,527
rac1^{-} + ***bsk***$^{-}$	2,23	2,07	0,513	0,620
rac1^{-} + ***rl***$^{-}$	2,22	2,17	0,520	0,553
rac1^{-} + ***puc***$^{-}$	2,07	1,88	0,620	0,747
**Puc Overexpression**	2,49	2,30	0,340	0,467

Modeling

To model the interaction network we first set a mathematical framework weighting up variations in protein activation, repression and protein expression.

Protein Activation and Repression

For a protein of concentration P, we pondered two different alternative activation scenarios, single or double activation inputs coming from one or two independent activators:

1) Single activation leads to two states of concentrations, P_0 (inactive) and P* (active), so

$$P_0 + P^* = P$$

Considering an activation input mediated by A and a repression input mediated by R,

$$dP^*/dt = A \, . \, P_0 - R \, . \, P^* = A \, . \, P - (A + R) \, . \, P^*$$

where A and R values integrate the linear reaction coefficients.

As the experimental FLIM data for the biosensor were acquired at equilibrium

$$dP^*/dt = 0$$

and then

$$P^*/P = A/(A+R) = (A/R)/(1+A/R)$$

with an activity ratio (χ)

$$\chi = \psi\ (A/R) \qquad (1)$$

being for each species (x)

$$\psi\ (x) = x/1+x$$

2) Double activation leads to three states of concentrations, P_0 (inactive) and P* and P**, so

$$P_0 + P^* + P^{**} = P$$

By considering A_1 and A_2 as activators leading respectively to P* and P** and R as a common repressor for both forms

$$dP^*/dt = A_1P_0 - RP^* = A_1(P - P^* - P^{**}) - RP^* = A_1(P - P^{**}) - (R + A_1)P^*$$

and

$$dP^{**}/dt = A_2P_0 - RP^{**} = A_2(P - P^* - P^{**}) - RP^{**} = A_2(P - P^*) - (R + A_2)P^{**}$$

At equilibrium

$$dP^*/dt = dP^{**}/dt = 0$$

so

$$\chi_1 = P^*/P\ = \psi\ (A_1/R)\ .\ (1 - P^{**}/P)$$

and

$$\chi_2 = P^{**}/P\ = \psi\ (A_2/R)\ .\ (1 - P^*/P)$$

then

$$\chi_1 = \psi\ (A_1/R)\ .\ [1- \psi\ (A_2/R)]/[1- \psi\ (A_1/R)\ .\ \psi\ (A_2/R)]$$

and

$$\chi_1 \approx \psi\ (A_1/R)\ .\ [1 - \psi\ (A_2/R)] \qquad (2)$$

Protein expression

We used the Michaelis-Menten formalism to describe the production of a protein P under the control of a transcription factor A,

$$dP/dt = V_{max}\ .\ \psi\ (A/K) - \delta\ .\ P$$

with

V_{max} the maximum velocity of protein production

K the Michaelis constant

δ the rate of protein degradation

At equilibrium

$$P = \nu \cdot \psi\,(A/K) \qquad (3)$$

with

$$\nu = V_{max}/\delta$$

Core dJun Network Assembly

Earlier described data indicate that in *Drosophila*, dJun (FRET biosensor) is activated (phosphorylated) by different kinase species with distinct affinities, Puc expression is induced as a result of Bsk activity, and Puc inhibits (dephosphorylates) Bsk (and other kinases). Importantly, this simple set of activities is challenged by the observed experimental results, in particular by the apparent repression of the FRET biosensor by Bsk. Thus, we search for novel interactions that could eventually fit the experimental data from single and double gene knockdowns.

We contemplated four species Bsk (JNK), Rl (ERK), ΣKin (an *ad-hoc* term integrating any other phosphorylation input in the biosensor, including other MAP kinases as P38s) and Puc (JNK dual-specificity Phosphatase). The topology of the network was compiled by deploying previously known interactions and implementing *ad hoc* in the model two new regulatory relationships: a positive loop mediated by Puc boosting Rl activity and an indirect repression of Puc expression resulting from an inhibitory activity of Rl on Bsk activity. Adopting these two new links, indirectly supported by the literature, let to precisely reproduce all experimental data (see below).

Considering A1, A2 and A3 as the concentrations of Bsk, Rl, and ΣKin, and Puc the concentration of Puc, their activities (kinases) according to (1) are

$$K_1 = A_1 \cdot \psi\,(\omega_1/Puc)$$

$$K_2 = A_2 \cdot \psi\,(\beta \cdot Puc^n) \cdot \psi\,(\omega_2/Puc) \qquad (4)$$

$$K_3 = A_3 \cdot \psi\,(\omega_3/Puc)$$

ω_1, ω_2, ω_3 and β are parameters to be determined by fitting. ω_i coefficients correspond to

$$\omega_i = \alpha_i/\kappa_i$$

with α_i the activation coefficient of the kinase and κ_i the affinity of Puc for each of the kinases. In each case, Puc acts as a repressor.

In equation (4), the positive loop mediated by Puc on Rl activity (see above) is modulated by the activation coefficient β. The power term (n) applied to Puc concentration describes an exponential amplification of the influence of Puc on Rl activity. In the fitting analysis, a value of 5 for the power term yielded the most accurate approximations.

In the modeled network, on the other hand, we hypothesized that Puc expression results from the conjunct activities of Bsk and Rl (see above). It has also been described that Fos, the Jun partner in the AP1 complex, can be phosphorylated by both Bsk and Rl resulting in different levels of regulatory transcriptional capabilities. Thus, we modeled Bsk and Rl interaction according to (2). So, Puc expression, using (3), is

$$\text{Puc} = \nu \,.\, \psi\,(K_1^{\,n} \,.\, [1 - \psi\,(K_2)]) \qquad (5)$$

ν is a constant parameter to be determined by fitting and denotes the ratio between the maximum speed of Puc production and its rate of degradation. The power term (n) applied to Bsk activity introduces cooperativity. In the fitting analysis, a value of 2 for the power term yielded the most accurate approximations to the experimental values of FRET.

Finally, we considered the DJun-FRET biosensor activation mediated indistinctly by all kinases

$$\text{FRET} = \psi\,(K_1 + K_2 + K_3) \qquad (6)$$

Implementation of the Mathematical Model

To solve equations (4) and (5), which are dependent on each other, we employed a numerical approach and applied the following algorithm

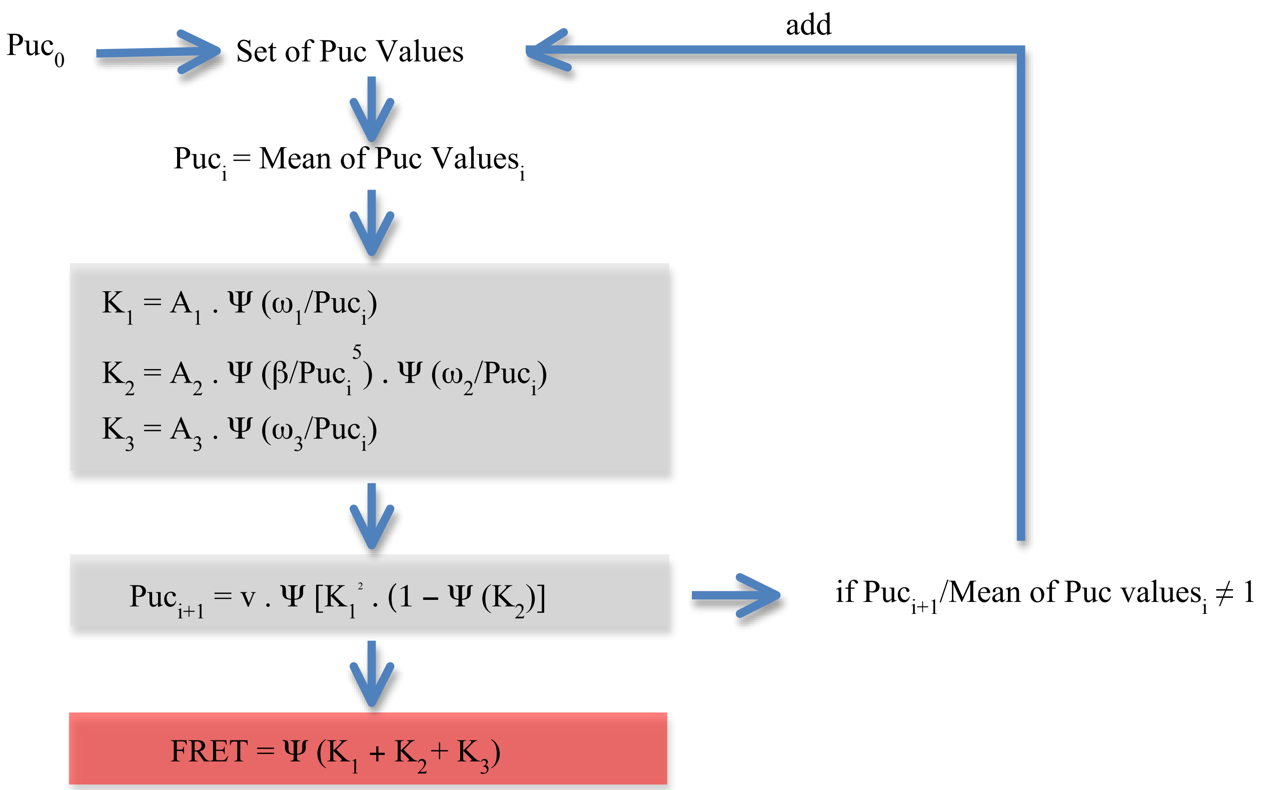

To solve the system we employed a known set of parameters $\{A_1, A_2, A_3, \omega_1, \omega_2, \omega_3, \beta,$ and $\nu\}$ and an initial value for Puc (Puc_0) feeding the loop determined for the WT condition at rest. We verified the convergence of the algorithm in all cases.

Fitting parameters

For each experimental condition at rest, namely WT, Bsk, Rl or Puc single and double knockdowns, we determined a common set of the parameters $\{A_1, A_2, A_3, \omega_1, \omega_2, \omega_3, \beta, \nu\}$ so that the modeled network recapitulates the measured FRET activity ratios. Additionally, three other parameters responding to the efficiencies of Puc (dPuc), Bsk (dBsk) and Rl (dRl) knockdowns were simulated as follow

Bsk knockdown: $A1 \leftarrow dBsk \, . \, A1$

Rl knockdown: $A2 \leftarrow dRl \, . \, A2$

Puc knockdown: $\nu \leftarrow dPuc \, . \, \nu$

Finally, we a-dimensioned the model by setting the Σ Kin concentration (A_3) to a constant value (1).

Upon stretch, we assumed that only the input parameters of the network ω_1, ω_2 and β are modulated taking the total number of independent parameters to 13 for 14 experimental FRET values.

Expanded Network

To determine the input of the Rac1 GTPase into the core dJun network, 8 experimental AR values were determined for the FRET biosensor in response to the knockdowns of Rac1, Rac1 + Bsk, Rac1 + Rl and Rac1 + Puc at rest and under stretch conditions. We presumed that Rac1 knockdown modulates the input parameters ω_1, ω_2, ω_3 and β by an equivalent factor, different *a priori* for each one, both at rest and under stretch conditions. So, we added 4 parameters for 8 new experimental FRET values.

To model the Puc overexpression, we added a term ν_o to the equation (5) of Puc expression, which simulates an independent production of the protein through the Gal4/UAS system. We used the parameter values found previously for the WT network, at rest or in stretched condition, to plot the FRET activation ratio against ν_o, which showed a unique value ν_o for a best fit.

Finally, the fit of the 22 experimental FRET values relies on 17 independent parameters.

Fitting algorithm

A Montecarlo algorithm was used to explore the parameter space to find a best fit with the FRET activities measured. We first fitted the parameters using the WT and knockdown experiments except the Rac1 knockdowns. Keeping these parameters, we then fitted the Rac1 knockdowns.
